# Compensating atmospheric adjustments reduce the volcanic forcing from Hunga stratospheric water vapor enhancement

**DOI:** 10.1126/sciadv.adl2842

**Published:** 2024-08-09

**Authors:** Yuwei Wang, Yi Huang

**Affiliations:** ^1^ Frontier Science Center for Deep Ocean Multispheres and Earth System (FDOMES) and Physical Oceanography Laboratory, Ocean University of China, Qingdao, China.; ^2^ Department of Atmospheric and Oceanic Sciences, McGill University, Montreal, Canada.

## Abstract

The 2022 eruption of the Hunga submarine volcano injected an unprecedented volume of water vapor into the stratosphere, presenting a unique, natural experiment for ascertaining the influence of stratospheric water vapor within the global radiation budget. This study examines the radiative forcings of the Hunga stratospheric water vapor enhancement, comparing stratosphere-adjusted radiative forcing derived from offline methods to an effective radiative forcing derived from Earth System Model simulations. Assuming a uniform 2 parts per million mass mixing ratio increase of water vapor in the Southern Hemisphere stratosphere, we estimated the instantaneous, stratosphere-adjusted, and overall effective radiative forcing to be −0.04, 0.08, and 0.05 W m^−2^, respectively. The lower magnitude of the positive volcanic stratospheric water vapor effective radiative forcing is due to compensating effects from atmospheric adjustments. Ensemble simulations of a coupled atmosphere-ocean model suggest a surface warming of 0.05 K, affirming a limited influence on global mean surface temperature from the volcanic stratospheric water vapor injection.

## Teaser

A systematic assessment is carried out to estimate the radiative forcings from Hunga stratospheric water vapor enhancement.

## INTRODUCTION

The submarine volcano Hunga Tonga-Hunga Ha’apai (to be referred as “Hunga” for brevity) progressed to its most explosive phase on 15 January 2022 (*1*). This event marked the most explosive eruption since Pinatubo in 1991, with a volcanic explosivity index of 5 (*2*). The plume’s overshooting top was observed above 50 km, setting a new altitude record in the satellite era (*3*, *4*). Distinct from conventional volcanos, a prominent characteristic of the Hunga eruption was volcanic emission of a very large amount of water vapor into the stratosphere, accompanied by relatively lower amounts of aerosols (*5*, *6*). The potential climate impacts of an explosive eruption tend predominantly to come from the amount of sulfur dioxide (SO_2_) emitted to the stratosphere. For instance, the Pinatubo eruption released 14 to 23 Tg of SO_2_ into the stratosphere (*7*). In contrast, the Hunga eruptions only injected 0.4 to 0.5 Tg of SO_2_ (*8*) but approximately 146 Tg of water vapor into the stratosphere, equivalent to roughly 10% of the total stratospheric water burden, providing a new case study for markedly different volcano-climate impacts (*5*, *9*).

The climate impact of the Hunga eruption has attracted considerable attention since the volcano erupted. Some early studies of the eruption’s climate effects primarily concentrated on the consequences arising from injected aerosols (*10*, *11*), ignoring the impact of the stratospheric water vapor (SWV) perturbation. These early inquiries consistently reported a negligible cooling effect, attributed to the modest burden of SO_2_ perturbation. However, a much higher than expected stratospheric aerosol optical depth (AOD) emerged in the weeks after the eruption, due largely to the coemitted water vapor sustaining SO_2_ oxidation for strengthened growth to optically active particle sizes (*12*). Also, because of a longer residence time of 5 to 10 years (*5*, *13*), SWV perturbation is postulated to be a substantial part of the eruption’s overall climate forcing.

Substantial progress has been made in understanding the influence of SWV on global mean surface temperature (GMST) in recent decades. Earlier research by Solomon *et al.* (*14*) found that changes in SWV could explain up to 25% of the GMST variation over the period 2000–2009. Subsequent studies mostly corroborated the warming effect of SWV within climate models (*15*–*17*). Prior studies have predominately investigated water vapor perturbation in the lower stratosphere in the context of global warming. However, the injection of water vapor into the middle stratosphere by the Hunga volcano raises questions about the impact of middle SWV on surface climate (*13*, *18*, *19*), although only a few studies have quantified the radiative forcing of Hunga SWV enhancement.

Here, we carry out a systematic assessment of the Hunga SWV forcing using the simulations of a radiative transfer model in offline mode and also a coupled atmosphere-ocean general circulation model. Our findings reveal that the effective radiative forcing (ERF) from Hunga SWV enhancement is reduced by compensating atmospheric adjustments, resulting in a limited surface climate response.

## RESULTS

There are three different metrics (Materials and Methods) used to quantify the magnitude of a climate relevant species’ radiative forcing: instantaneous radiative forcing (IRF), stratospheric-adjusted radiative forcing (SARF), and ERF. For IRF, we apply the rapid radiative transfer model for general circulation models (RRTMG) (*20*), integrated into the Community Earth System Model (CESM) (*21*), to quantify the IRF resulting from the Hunga eruption. To approximately represent the enhancement from Hunga, we introduce a perturbation of SWV into the model, within the Southern Hemisphere (SH). The perturbation is 2 parts per million (ppm) mass mixing ratio (MMR) of H_2_O added to the background climatology state between 45 and 10 hPa in the middle stratosphere and between 60°S and 0°. Within this domain, a 2 ppm MMR increase is equivalent to a total injection of approximately 158 Tg of H_2_O mass, which corresponds closely with the SWV increase observed from the microwave limb sounder (MLS)–Aura satellite observations (*5*). The SWV perturbation yields an IRF of 0.08 W m^−2^ at the tropopause, as detailed in Table 1. This effect is primarily attributed to the enhanced radiative emissivity of the stratosphere. Note that at the top of the atmosphere (TOA), the IRF is −0.04 W m^−2^. This is different from other greenhouse gases such as CO_2_, which typically yield positive IRFs at both TOA and tropopause. The negative TOA IRF of SWV results from the moistening at very high altitudes, which, due to the temperature inversion in the stratosphere, can lead to a slight increase of outgoing longwave radiation and thus a negative forcing, in consistency with the sign of water vapor kernels (*22*) in the middle stratosphere.

**Table 1.** Radiative forcing (W m^−2^). IRF, instantaneous radiative forcing; SARF, stratospheric-adjusted radiative forcing; ERF, effective radiative forcing, mean ± SE.

	IRF	SARF	ERF
TOA	−0.04	0.08	0.05 ± 0.25
Tropopause	0.08	0.08	0.05 ± 0.25
Surface	0.00	0.00	0.06 ± 0.28

**Fig. 1.**
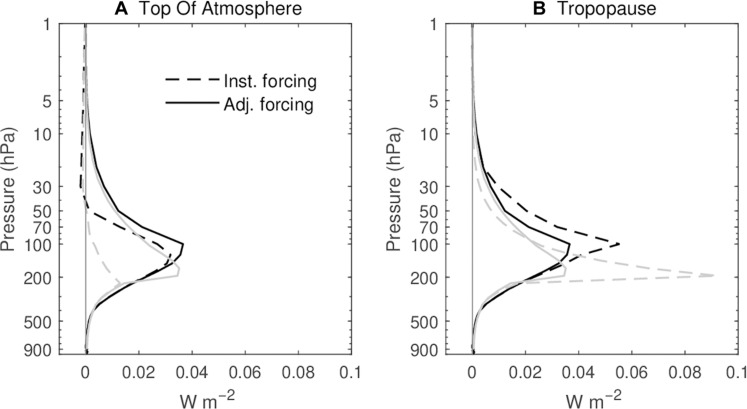
The radiative effects of 1 km-depth layered SWV enhancements at different altitudes on radiative forcing calculated through a radiative transfer model. The calculations are based on the monthly mean profile of January 2022 at Hunga location with tropopause at 100 hPa (black lines) and U.S. standard atmospheric profile with tropopause at 200 hPa (gray lines) for a uniform change of 1 ppmv of water vapor in 1-km layers. Dashed lines represent the instantaneous radiative forcing. The solid lines represent SARF. Panel (**A**) profiles showing IRF and SARF at the top of the atmosphere for each altitude’s 1-km layered SWV enhancement, and panel (**B**) for the two forcings at the tropopause.

Figure 1 illustrates how the radiative forcing from a layered SWV enhancement depends strongly on its altitude. Specifically, the profiles in the figure show the forcing at the TOA (Fig. 1A) and the tropopause (Fig. 1B) for a 1 part per million volume (ppmv) in 1-km depth layer at different vertical levels, calculated with RRTMG. The calculations are based on January 2022 monthly mean atmospheric profile at Hunga location derived from the European Centre for Medium-Range Weather Forecasts (ECMWF) Reanalysis v5 (ERA5) dataset. The profile has 37 levels, with the top level at 1 hPa and tropopause at 100 hPa. At TOA, the negative IRF (dashed black line in Fig. 1A) is observed in the levels above 50 hPa, where the Hunga-injected water vapor is predominately located. The IRF at the tropopause (dashed black line in Fig. 1B) is larger in magnitude than that at the TOA. Water vapor near the tropopause demonstrates the highest sensitivity, consistent with the findings in previous studies (*14*, *23*). A standard profile for the U.S. location (gray lines), with tropopause at 200 hPa, is also used to represent the typical profile of mid-latitudes. The result from the U.S. profile is consistent with that at Hunga location, both demonstrating the negative TOA IRF above 50 hPa and highest sensitivity near the tropopause.

**Fig. 2.**
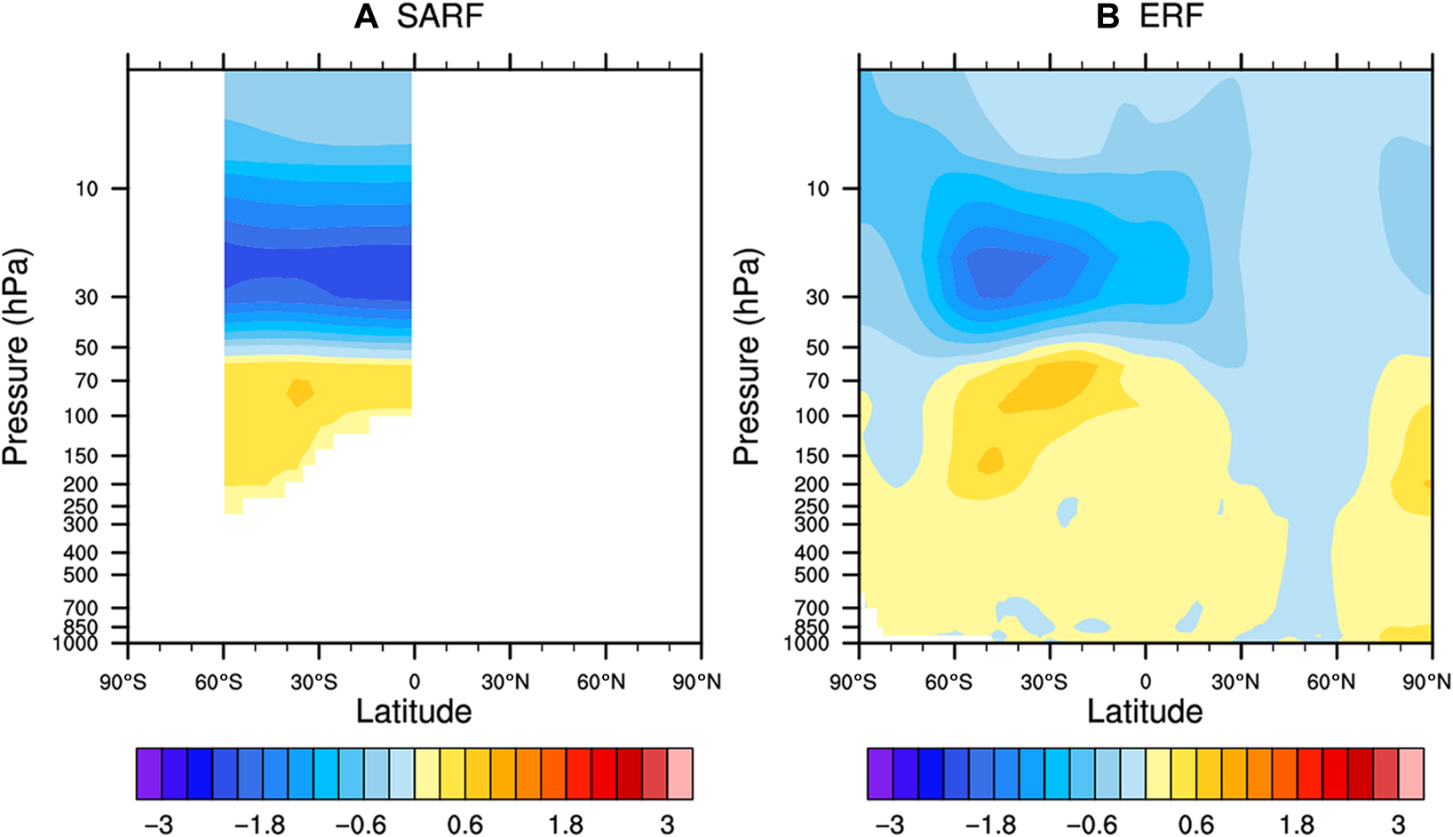
Atmospheric temperature response to SWV enhancement of 2 ppm of MMR between 45 and 10 hPa in altitude and 60°S and 0° in latitude. (**A**) SARF experiment, in which the stratosphere radiatively relaxes back to an equilibrium state. (**B**) Effective radiative forcing experiment, in which both the stratosphere and troposphere relax to equilibrium, while the sea surface temperature is fixed. Units: K.

Having explored the idealized radiative forcing of global 1-km layered SWV perturbations at different altitudes, here, we impose a deeper global SWV enhancement to approximately represent the excess 150 Tg of water vapor the Hunga eruption added to the stratosphere. We used the same model (RRTMG) to estimate the SARF. The injection of SWV intensifies the longwave cooling rate in the middle stratosphere. Consequently, the temperature in the middle and upper stratosphere decreases, resulting in a reduction in its thermal emission (*24*, *25*). This decrease continues until a radiative equilibrium is reestablished, restoring the energy balance in the stratosphere. The temperature decrease associated with the deeper Hunga-like SWV perturbation spans the entire region, extending from approximately 60 hPa to the TOA, with the maximum center around 20 hPa (Fig. 2A). Correspondingly, the temperature in the lower stratosphere increases, albeit in a smaller region and with a smaller magnitude. The stratospheric cooling gives rise to an additional positive radiative forcing at the TOA. Our calculations yield a SARF value of 0.08 W m^−2^ at both the TOA and the tropopause. The magnitude of SARF well aligns with the IRF at the tropopause, indicating that the tropopause IRF is a good approximation of SARF for Hunga SWV perturbation. The solid lines in Fig. 1 display the profiles of the H_2_O kernel including the stratospheric adjustment. At tropopause, the SARF closely matches the IRF above 45 hPa, consistent with the result of Hunga SWV enhancement.

The quantification of the ERF requires to consider how the troposphere and stratosphere adjust to the Hunga-like SWV enhancement. To partially address this requirement, we applied the Community Atmospheric Model 5 (CAM5) (*21*), which has a comprehensive representation of tropospheric processes, but with prescribed stratospheric ozone layer and a 30-level vertical resolution (Materials and Methods). The SWV cooling of the stratosphere causes a slowdown of the Brewer-Dobson circulation (*26*), and a future study could assess also how the resulting stratospheric ozone layer responses (*27*) could also be relevant for the eruption’s ERF. Model simulations reveal cooling in the middle and upper stratosphere and warming in the lower stratosphere (Fig. 2B), consistent with the findings from the offline SARF radiative transfer calculations (Fig. 2A). With this version of CESM, the model troposphere responds to the stratospheric post-Hunga SWV increase with a warming effect, with notable changes in high cloud. These tropospheric adjustments are found in the model to emerge rapidly (in the first month) in the simulation and yield an ERF value of 0.05 W m^−2^, which is approximately only half of both the SARF and the IRF. This finding is in contrast with the assumption made in (*13*), where they assumed a larger magnitude of the forcing equivalent to the value of IRF. Our results indicate that this assumption overestimates the actual forcing and thus likely the climate impact of the SWV perturbation.

To identify the specific tropospheric processes that offset the SARF, we use the radiative kernels (Materials and Methods) to decompose the flux perturbations at the TOA (Table 2). Our analysis reveals that the most negative compensation arises from tropospheric warming, contributing −0.069 W m^−2^ to the TOA flux. Following tropospheric warming, the second contributor is the decrease in high cloud cover, which reduces the TOA forcing by −0.048 W m^−2^.

**Table 2.** TOA radiative flux decomposition (W m^−2^) in ERF experiment. dR, radiative flux anomaly; TOA, top of atmosphere; Tstra, stratospheric temperature; Ttrop, tropospheric temperature; WVstra, stratospheric water vapor; WVtrop, tropospheric water vapor; TS, surface temperature; Alb, surface albedo; Cld: cloud; Res: residual.

dR_TOA	dR_Tstra	dR_Ttrop	dR_WVstra	dR_WVtrop	dR_TS	dR_Alb	dR_Cld	dR_Res
0.049	0.138	−0.069	−0.027	0.060	−0.012	0	−0.048	0.008

We conducted another simulation to test the sensitivity of the radiative forcing to the horizontal spread of the water vapor plume. In this simulation, we perturbed the SWV globally by adding 1 ppm MMR of water vapor to the climatology between 45 and 10 hPa and 60°S and 60°N. The middle and upper stratospheric cooling and lower stratospheric warming consistently occur globally in SARF and ERF (fig. S1). The magnitudes of IRF, SARF, and ERF are nearly identical to the SH-only SWV-increase simulations (table S1). The TOA radiative flux decompositions consistently reveal the compensating atmospheric adjustments for the ERF (table S2).

To provide a context for appreciating the SWV ERF value of 0.05 W m^−2^, we can translate it into an equivalent amount of CO_2_ perturbation for comparison. The ERF associated with a quadrupling of CO_2_, as estimated from a large ensemble of Phase 6 of the Coupled Model Intercomparison Project (CMIP6) models, is approximately 7.98 W m^−2^ (*28*). Therefore, the ERF resulting from the SWV perturbation is equivalent to approximately 0.6% of the ERF resulting from a quadrupling of CO_2_ or equivalently about 2.6 ppm perturbation of the CO_2_ concentration based on the logarithmic dependence of its forcing magnitude on its concentration (*29*, *30*). Thus, the ERF resulting from the SWV injection due to the Hunga volcanic eruption is approximately equivalent to the increase in CO_2_ concentration over 1 year.

**Fig. 3.**
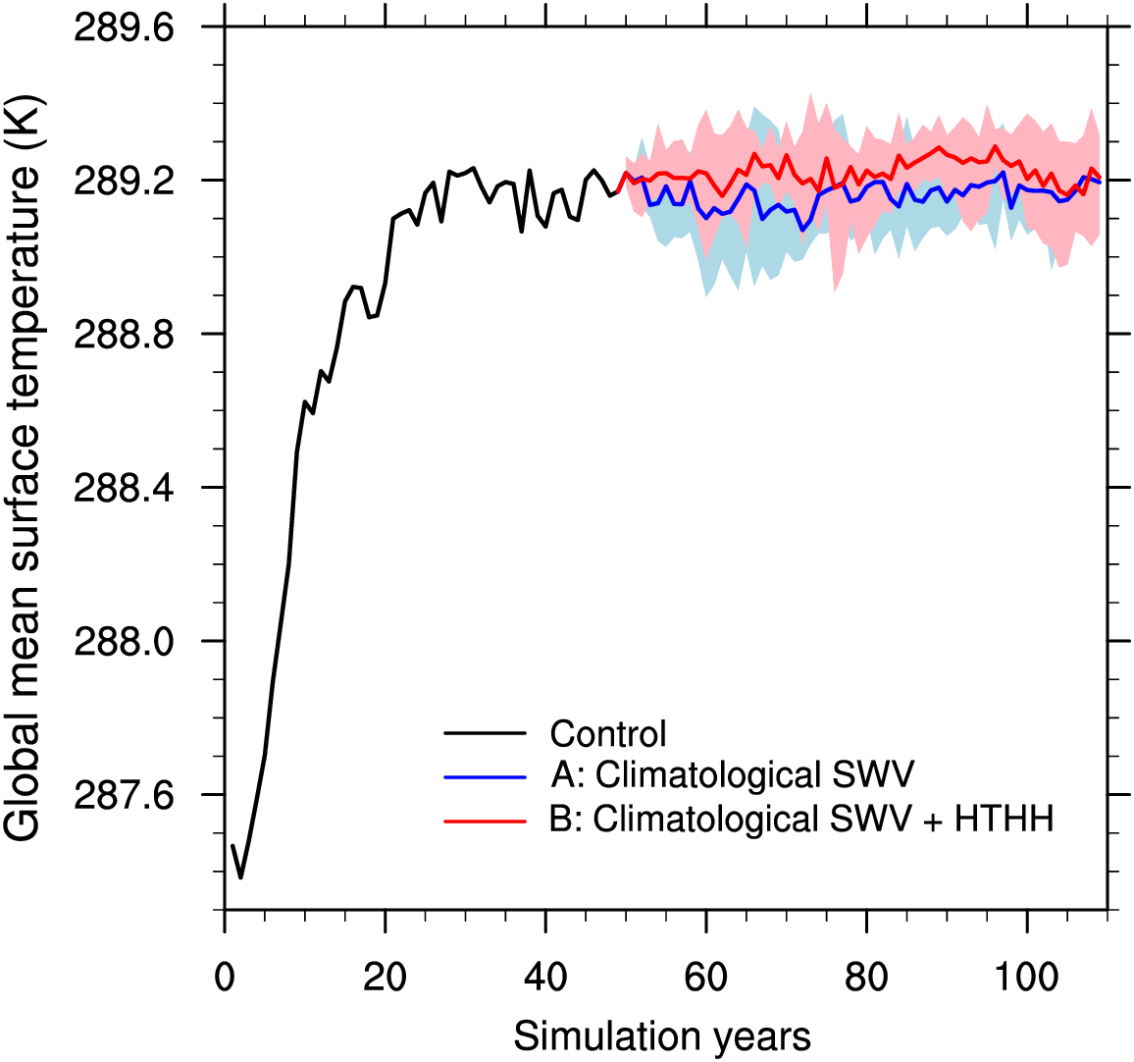
Temporal evolution of the global annual mean surface temperature. The solid black line represents the spin-up control run. Experiments A and B extend from the control run with different prescribed SWV concentrations. Experiment A is driven by climatological SWV concentration, while experiment B incorporates the increase of SWV from Hunga volcanic eruption. The SWV increase is prescribed in a region bounded by 45 and 10 hPa and between 60°S and 60°N. Experiments A and B consist of five members, respectively. The shading indicates the range of temperature by the five ensemble members.

To affirm the climate impacts of the SWV injection, we use the coupled atmosphere-ocean model CESM to directly assess the surface climate response to the Hunga eruption. Given the transient nature of the SWV forcing associated with the Hunga eruption, we adopt a “slab ocean” configuration, wherein ocean circulation is prescribed, and only the ocean thermodynamics are taken into account. We began by running the model for 50 years to reach an equilibrium state. Following this initial phase, we continued the model run with different SWV configurations for our experiments, labeled with A and B, respectively. In experiment A, we prescribed monthly climatological water vapor in the middle stratosphere. In experiment B, we prescribed the climatological SWV along with the SWV perturbation resulting from the Hunga eruption (Materials and Methods). Both experiments consisted of five ensemble members and ran for an additional 60 years, and we analyzed the data from the last 30 years of each run. Simulations indicate that the GMST in experiment B increased by 0.05 K relative to experiment A (Fig. 3). This suggests that the SWV injection of Hunga eruption has a relatively minor impact on the GMST. This finding is consistent with the more conservative estimations of the climate impact of Hunga (*12*, *18*, *26*).

## DISCUSSION

Our study used a combination of a radiative transfer model and an atmospheric general circulation model to evaluate the radiative forcing and climate impacts of the SWV injected by the Hunga volcanic eruption. Assuming a 2 ppm MMR of uniform SWV enhancement in SH’s middle stratosphere, our calculations quantify the IRF, SARF, and ERF at TOA to be −0.04, 0.08, and 0.05 W m^−2^ and at tropopause to be 0.08, 0.08, and 0.05 W m^−2^. The reduction in the ERF compared to the stratosphere-adjusted forcing is due to the compensating tropospheric adjustments. Ensemble simulations conducted using the coupled atmosphere-ocean model suggest that the GMST change attributable to the stratospheric water increase is 0.05 K. Our findings suggest that the SWV increase resulting from the Hunga volcanic eruption has a limited effect on GMST.

An important consideration in our calculation is that we assume a constant SWV perturbation, matching the value retrieved from satellite data immediately following the Hunga eruption. Schoeberl *et al.* (*25*) have quantified radiative flux changes using the observed SWV changes from the MLS satellite instrument and found that the water vapor forcing was transient and decreased by more than half from the peak in mid-April to the value in mid-December. Therefore, the radiative forcing and surface temperature response quantified here presents the upper limit for the climate impact of the Hunga eruption.

One robust feature in our simulations is the stratospheric cooling, centered at 20 hPa. This anomalous cooling was also identified in the MLS satellite data (*31*) and quantified from the data assimilation system analysis increments (*26*). Our simulation, which confines the water vapor between 0° and 60°S, reproduced a comparable magnitude of cooling to the observations (Fig. 1), with the maximum exceeding −2 K. However, our simulation reveals a cooling extending to higher altitudes, along with an additional warming in the lowermost stratosphere, which is not evident in the observations. One factor influencing the stratospheric temperature but not included in our simulation is the quasi-biennial oscillation (QBO). The observed stratospheric temperature change arises from both the QBO and the forcing from the volcano eruption (*32*).

We focused on the effects of the enhanced SWV from the Hunga eruption, not including the aerosols due to their small burden. However, a recent study (*25*) suggested that the negative radiative forcing of the volcanic aerosols from Hunga may exceed the positive SWV radiative forcing as the eruption-induced AOD may be higher than what is expected from the limited SO_2_ emissions (*6*, *12*), which may further limit the surface warming effect of the Hunga eruption.

Note that this study is based on a model with relatively coarse stratospheric resolution and prescribed stratospheric ozone, which potentially affects the forcing estimate. Nevertheless, the reduced ERF of 0.05 W m^−2^ results from compensating atmosphere adjustments. The importance of these compensation effects as evidenced here warrants further research to investigate the adjustment processes more comprehensively, for example, using models with higher stratospheric vertical resolution and interactive ozone layer chemistry.

## MATERIALS AND METHODS

### GCM setup and SWV locking configuration

The GCM model used in this study is CESM 1.2 (*21*). The atmospheric component within the model is CAM5. The model is set up with a horizontal resolution of 1.9° × 2.5° and encompasses 30 levels in the vertical direction. The vertical coordinate is a hybrid sigma-pressure system. The upper regions of the atmosphere, where water vapor was injected by Hunga volcanic eruptions, are discretized on pressure alone. The radiative transfer parameterization implemented in CAM5 is the RRTMG (*20*) scheme.

We conducted a CESM simulation for 50 years and used it as the control climate. The model achieved an equilibrium state approximately after 30-year integrations. To create a dataset for climatological water vapor, we archived the monthly mean water vapor concentrations from the final 10 years of the simulation.

We continued the simulation with three prescribed SWV concentrations, labeled with experiment A, experiment B, and experiment C, respectively. In experiment A, we used the archived climatological SWV values. Experiment B involved the prescription of climatological SWV values augmented by an additional 1 ppm MMR of water vapor in stratosphere, spanning from an altitude of 10 to 45 hPa and ranging in latitudes from 60°S to 60°N. The 1 ppm MMR increase in water vapor is roughly equivalent to SWV perturbation resulting from the Hunga volcanic eruption. All other configurations are the same in experiments A and B. Experiment C is similar to experiment B except that the SWV enhancement is confined in SH, with 2 ppm MMR of water vapor between 10 and 45 hPa in altitude and 60°S to 0° in latitude.

To prevent the introduction of artificial sources and sinks of water vapor in the stratosphere and to highlight the radiative forcing of the SWV perturbation, our modifications were confined to the radiation scheme (*17*, *33*). The prescribed SWV was interpolated vertically to adapt to the varying pressure at each hybrid level and temporally to fit the specific time step, ensuring that the SWV distribution was appropriately represented in the model.

### SWV radiative forcing calculation

Three SWV radiative forcing values are calculated in this work: (i) IRF, (ii) SARF, and (iii) ERF. IRF is defined as the instantaneous change in energy flux resulting from a change in the forcing agent. For SARF, the stratosphere is relaxed back to an energy-balanced state, leaving only energy imbalance in the troposphere. After the stratospheric adjustment, the change in energy flux at the tropopause is equal to that at the TOA. The concept of SARF is widely used in Intergovernmental Panel on Climate Change (IPCC) Assessment Reports, as it is more accurate than the IRF for predicting surface climate change and the calculation is not difficult to implement. Not including tropospheric processes, SARF can exhibit biases in predicting the surface temperature response (*34*). Thus, the inclusion of rapid tropospheric adjustments has the advantage of providing a more accurate prediction. Previous studies demonstrated that ERF generally performs the best in predicting climate responses (*35*).

The estimations of IRF and SARF are accomplished through an online radiative transfer calculation using a double radiation call scheme. In this scheme, at each time step of CAM integration, the radiation scheme is invoked twice: once for the default climate simulation and then for the perturbed climate simulation (*36*). In the context of the Hunga eruption, the distinction between these two radiative transfer calls lies in the concentration of SWV.

The quantification of the ERF is achieved through a prescribed sea surface temperature (SST) simulation, conducted using the CAM5. To facilitate this simulation, climatological monthly mean SSTs and sea ice concentrations from the control experiment are archived. These archived data are then used to drive the prescribed SST experiment. The simulations are run continuously for 30 years. The ERF is calculated as the average over the final 20 years of the simulation.

### Radiative kernel

The radiative kernel method is used to quantify the TOA radiative flux change caused by the perturbation or response in the atmospheric constituents. The change in TOA flux is estimated as ∆*R_i_* = *k_i_* · ∆*X_i_*, where ∆*X_i_* is the change in atmospheric components associated with energy perturbation. The radiative kernel *k_i_* is the TOA radiation sensitivity to the climate variable *X_i_*. The radiative kernel used in this study is based on (*22*).

## References

[1] R. S. Matoza, D. Fee, J. D. Assink, A. M. Iezzi, D. N. Green, K. Kim, L. Toney, T. Lecocq, S. Krishnamoorthy, J.-M. Lalande, Atmospheric waves and global seismoacoustic observations of the January 2022 Hunga eruption, Tonga. Science 377, 95–100 (2022). 35549311 10.1126/science.abo7063

[2] Hunga Tonga–Hunga Ha’apai (Smithsonian Institution, 2022); https://volcano.si.edu/volcano.cfm?vn=243040.

[3] J. L. Carr, Á. Horváth, D. L. Wu, M. D. Friberg, Stereo plume height and motion retrievals for the record-setting Hunga Tonga-Hunga Ha’apai eruption of 15 January 2022. Geophys. Res. Lett. 49, e2022GL098131 (2022).

[4] S. R. Proud, A. T. Prata, S. Schmauß, The January 2022 eruption of Hunga Tonga-Hunga Ha’apai volcano reached the mesosphere. Science 378, 554–557 (2022). 36378963 10.1126/science.abo4076

[5] L. Millan, M. L. Santee, A. Lambert, N. J. Livesey, F. Werner, M. J. Schwartz, H. C. Pumphrey, G. L. Manney, Y. Wang, H. Su, The Hunga Tonga-Hunga Ha’apai hydration of the stratosphere. Geophys. Res. Lett. 49, e2022GL099381 (2022). 10.1029/2022GL099381 PMC9285945 35865735

[6] B. Legras, C. Duchamp, P. Sellitto, A. Podglajen, E. Carboni, R. Siddans, J.-U. Grooß, S. Khaykin, F. Ploeger, The evolution and dynamics of the Hunga Tonga–Hunga Ha’apai sulfate aerosol plume in the stratosphere. Atmosp. Chem. Phys. 22, 14957–14970 (2022).

[7] S. Guo, G. J. Bluth, W. I. Rose, I. M. Watson, A. Prata, Re-evaluation of SO2 release of the 15 June 1991 Pinatubo eruption using ultraviolet and infrared satellite sensors. Geochem. Geophys. Geosyst. 5, 654 (2004).

[8] S. Carn, N. Krotkov, B. Fisher, C. Li, Out of the blue: Volcanic SO2 emissions during the 2021–2022 eruptions of Hunga Tonga—Hunga Ha’apai (Tonga). Front. Earth Sci. 10, 976962 (2022).

[9] H. Vömel, S. Evan, M. Tully, Water vapor injection into the stratosphere by Hunga Tonga-Hunga Ha’apai. Science 377, 1444–1447 (2022). 36137033 10.1126/science.abq2299

[10] H. Zhang, F. Wang, J. Li, Y. Duan, C. Zhu, J. He, Potential impact of Tonga Volcano eruption on global mean surface air temperature. J. Meteorol. Res. 36, 1–5 (2022).

[11] M. Zuo, T. Zhou, W. Man, X. Chen, J. Liu, F. Liu, C. Gao, Volcanoes and climate: Sizing up the impact of the recent Hunga Tonga-Hunga Ha’apai volcanic eruption from a historical perspective. Adv. Atmosp. Sci. 39, 1986–1993 (2022).

[12] Y. Zhu, C. G. Bardeen, S. Tilmes, M. J. Mills, X. Wang, V. L. Harvey, G. Taha, D. Kinnison, R. W. Portmann, P. Yu, Perturbations in stratospheric aerosol evolution due to the water-rich plume of the 2022 Hunga-Tonga eruption. Commun. Environ. 3, 248 (2022).

[13] S. Jenkins, C. Smith, M. Allen, R. Grainger, Tonga eruption increases chance of temporary surface temperature anomaly above 1.5 °C. Climate Change 13, 127–129 (2023).

[14] S. Solomon, K. H. Rosenlof, R. W. Portmann, J. S. Daniel, S. M. Davis, T. J. Sanford, G.-K. Plattner, Contributions of stratospheric water vapor to decadal changes in the rate of global warming. Science 327, 1219–1223 (2010). 20110466 10.1126/science.1182488

[15] A. Dessler, M. Schoeberl, T. Wang, S. Davis, K. Rosenlof, J. P. Vernier, Variations of stratospheric water vapor over the past three decades. J. Geophys. Res. Atmos. 119, 12588–12598 (2014).

[16] A. Banerjee, G. Chiodo, M. Previdi, M. Ponater, A. J. Conley, L. M. Polvani, Stratospheric water vapor: An important climate feedback. Climate Dynam. 53, 1697–1710 (2019).

[17] Y. Wang, Y. Huang, The surface warming attributable to stratospheric water vapor in CO2-caused global warming. J. Geophys. Res. Atmos. 125, e2020JD032752 (2020).

[18] Y. Bao, Y. Song, Q. Shu, Y. He, F. Qiao, Tonga volcanic eruption triggered anomalous Arctic warming in early 2022. Ocean Model. 186, 102258 (2023).

[19] S. D. Guzewich, L. D. Oman, P. R. Colarco, J. A. Richardson, P. L. Whelley, T. J. Fauchez, R. K. Kopparapu, S. T. Bastelberger, A potential surface warming regime for volcanic super‐eruptions through stratospheric water vapor increases. J. Geophys. Res. Atmos. 129, e2023JD038667 (2024).

[20] E. J. Mlawer, S. J. Taubman, P. D. Brown, M. J. Iacono, S. A. Clough, Radiative transfer for inhomogeneous atmospheres: RRTM, a validated correlated-k model for the longwave. J. Geophys. Res. Atmos. 102, 16663–16682 (1997).

[21] J. W. Hurrell, M. M. Holland, P. R. Gent, S. Ghan, J. E. Kay, P. J. Kushner, J.-F. Lamarque, W. G. Large, D. Lawrence, K. Lindsay, The community earth system model: A framework for collaborative research. Bull. Am. Meteorol. Soc. 94, 1339–1360 (2013).

[22] Y. Huang, Y. Xia, X. Tan, On the pattern of CO_2_ radiative forcing and poleward energy transport. J. Geophys. Res. Atmos. 122, 10578–10593 (2017).

[23] A. C. Maycock, M. M. Joshi, K. P. Shine, A. A. Scaife, The circulation response to idealized changes in stratospheric water vapor. J. Climate 26, 545–561 (2013).

[24] A. C. Maycock, K. P. Shine, M. M. Joshi, The temperature response to stratospheric water vapour changes. Q. J. Roy. Meteorol. Soc. 137, 1070–1082 (2011).

[25] M. R. Schoeberl, Y. Wang, R. Ueyama, A. Dessler, G. Taha, W. Yu, The estimated climate impact of the Hunga Tonga-Hunga Ha’apai eruption plume. Geophys. Res. Lett. 50, e2023GL104634 (2023).

[26] L. Coy, P. A. Newman, K. Wargan, G. Partyka, S. Strahan, S. Pawson, Stratospheric circulation changes associated with the Hunga Tonga-Hunga Ha’apai eruption. Geophys. Res. Lett. 49, e2022GL100982 (2022).

[27] X. Wang, W. Randel, Y. Zhu, S. Tilmes, J. Starr, W. Yu, R. Garcia, O. B. Toon, M. Park, D. Kinnison, Stratospheric climate anomalies and ozone loss caused by the Hunga Tonga-Hunga Ha’apai volcanic eruption. J. Geophys. Res. Atmos. 128, e2023JD039480 (2023).

[28] C. J. Smith, R. J. Kramer, G. Myhre, K. Alterskjær, W. Collins, A. Sima, O. Boucher, J.-L. Dufresne, P. Nabat, M. Michou, Effective radiative forcing and adjustments in CMIP6 models. Atmosp. Chem. Phys. 20, 9591–9618 (2020).

[29] M. Etminan, G. Myhre, E. J. Highwood, K. P. Shine, Radiative forcing of carbon dioxide, methane, and nitrous oxide: A significant revision of the methane radiative forcing. Geophys. Res. Lett. 43, 12614–12623 (2016).

[30] Y. Huang, M. Bani Shahabadi, Why logarithmic? A note on the dependence of radiative forcing on gas concentration. J. Geophys. Res. Atmos. 119, 13683–13689 (2014).

[31] M. R. Schoeberl, Y. Wang, R. Ueyama, G. Taha, E. Jensen, W. Yu, Analysis and impact of the Hunga Tonga-Hunga Ha’apai stratospheric water vapor plume. Geophys. Res. Lett. 49, e2022GL100248 (2022).

[32] J. Angell, Stratospheric warming due to Agung, El Chichón, and Pinatubo taking into account the quasi-biennial oscillation. J. Geophys. Res. Atmos. 102, 9479–9485 (1997).

[33] Y. Huang, Y. Wang, H. Huang, Stratospheric water vapor feedback disclosed by a locking experiment. Geophys. Res. Lett. 47, e2020GL087987 (2020).

[34] J. Hansen, M. Sato, R. Ruedy, L. Nazarenko, A. Lacis, G. Schmidt, G. Russell, I. Aleinov, M. Bauer, S. Bauer, Efficacy of climate forcings. J. Geophys. Res. Atmos. 110, 5776 (2005).

[35] T. Richardson, P. Forster, C. Smith, A. Maycock, T. Wood, T. Andrews, O. Boucher, G. Faluvegi, D. Fläschner, Ø. Hodnebrog, Efficacy of climate forcings in PDRMIP models. J. Geophys. Res. Atmos. 124, 12824–12844 (2019). 32025453 10.1029/2019JD030581 PMC6988499

[36] Y. Wang, Y. Huang, Understanding the atmospheric temperature adjustment to CO2 perturbation at the process level. J. Climate 33, 787–803 (2020).

