# Supplementary material for: Compensating atmospheric adjustments reduce the volcanic forcing from Hunga stratospheric water vapor enhancement
Source: Sci Adv. 2024 Aug 9;10(32):eadl2842. doi: 10.1126/sciadv.adl2842 (PMC11313849; doi:10.1126/sciadv.adl2842)
Supplement: Supplementary file 1 — Fig. S1 Tables S1 and S2 [file sciadv.adl2842_sm.pdf]

Science Advances
AAAS

Supplementary Materials for

Compensating atmospheric adjustments reduce the volcanic forcing from Hunga stratospheric water vapor enhancement

Yuwei Wang and Yi Huang

Corresponding author: Yuwei Wang, wyw@ouc.edu.cn; Yi Huang, yi.huang@mcgill.ca

Sci. Adv. **10**, eadl2842 (2024)
DOI: 10.1126/sciadv.adl2842

This PDF file includes:

Fig. S1
Tables S1 and S2

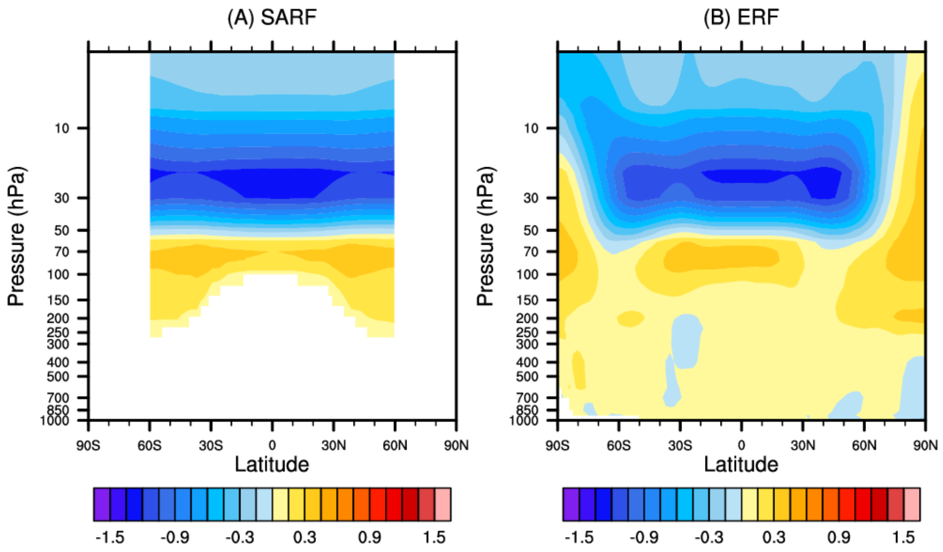

Fig. S1. Atmospheric temperature response to stratospheric water vapor enhancement of 1 ppm mass mixing ratio between 45 and 10 hPa in altitude and 60 °S and 60 °N in latitude. (a) Stratosphere-adjusted radiative forcing experiment, in which the stratosphere radiatively relaxes back to an equilibrium state. (b) Effective radiative forcing experiment, in which both the stratosphere and troposphere relax to equilibrium, while the sea surface temperature is fixed. Units: K.

Table S1. Radiative Forcing (W m^{-2}) for Stratospheric Water Vapor Enhancement with Global Symmetric Distribution

	IRF	SARF	ERF
TOA	-0.04	0.08	0.05±0.30
Tropopause	0.09	0.08	0.05±0.30
Surface	0.00	0.00	0.05±0.32

IRF: Instantaneous Radiative Forcing

SARF: Stratospherically Adjusted Radiative Forcing

ERF: Effective Radiative Forcing, Mean ± Standard Error

Stratospheric water vapor perturbation is 1ppm mass mixing ratio added between 45 and 10 hPa in altitude and 60 °S and 60 °N in latitude.

Table S2. TOA Radiative Flux Decomposition ($W\ m^{-2}$) in ERF Experiments with Globally Symmetric Stratospheric Water Vapor Enhancement

dR_TOA	dR_Tstra	dR_Ttrop	dR_WVstra	dR_WVtrop	dR_TS	dR_Alb	dR_Cld	dR_Res
0.048	0.17	**-0.083**	-0.032	0.069	-0.018	0	**-0.02**	-0.034

dR: Radiative flux anomaly; TOA: Top of atmosphere; Tstra: Stratospheric temperature; Ttrop: Tropospheric temperature; WVstra: Stratospheric water vapor; WVtrop: Tropospheric water vapor; TS: Surface temperature; Alb: Surface albedo; Cld: Cloud; Res: Residual
